# Transgenerational exposure to marine heatwaves ameliorates the lethal effect on tropical copepods regardless of predation stress

**DOI:** 10.1002/ece3.9149

**Published:** 2022-08-04

**Authors:** Kiem N. Truong, Ngoc‐Anh Vu, Nam X. Doan, Canh V. Bui, Minh‐Hoang Le, Minh T. T. Vu, Khuong V. Dinh

**Affiliations:** ^1^ Department of Ecology University of Science, Vietnam National University Hanoi Vietnam; ^2^ Cam Ranh Centre for Tropical Marine Research and Aquaculture, Institute of Aquaculture Nha Trang University Nha Trang City Vietnam; ^3^ Section for Aquatic Biology and Toxicology, Department of Biosciences University of Oslo Oslo Norway

**Keywords:** biotic interaction, climate change, coastal ecosystems, predator–prey interaction, transgenerational acclimation

## Abstract

Marine heatwaves (MHWs) are emerging as a severe stressor in marine ecosystems. Extreme warm sea surface temperatures during MHWs often exceed the optimal thermal range for more than one generation of tropical coastal zooplankton. However, it is relatively unknown whether transgenerational plasticity (TGP) to MHWs may shape the offspring's fitness, particularly in an ecologically relevant context with biotic interactions such as predation stress. We addressed these novel research questions by determining the survival, reproductive success, and grazing rate of the copepod *Pseudodiaptomus incisus* exposed to MHW and fish predator cues (FPC) for two generations (F1 and F2). The experiment was designed in a full orthogonal manner with 4 treatments in F1 and 16 treatments in F2 generation. In both generations, MHW reduced *P. incisus* survival, reproductive parameters, and grazing by 10%–62% in MHW, but these parameters increased by 2%–15% with exposure to FPC, particularly at control temperature. F2 reproductive success and grazing rate as indicated by cumulative fecal pellets were reduced by 20%–30% in F1‐MHW, but increased by ~2% in F1‐FPC. Strikingly, MHW exposure reduced 17%–18% survival, but transgenerational exposure to MHWs fully ameliorated its lethal effect and this transgenerational effect was independent of FPC. Increased survival came with a cost of reduced reproductive success, constrained by reduced grazing. The rapid transgenerational MHW acclimation and its associated costs are likely widespread and crucial mechanisms underlying the resilience of coastal tropical zooplankton to MHWs in tropical coastal marine ecosystems.

## Graphical abstract

Marine heatwaves emerge as a severe stressor in marine ecosystems, yet it is relatively unknown whether transgenerational effect of marine heatwaves may shape the offspring fitness, particularly in an ecologically relevant context with biotic interactions such as predation stress. Our study showed that transgenerational exposure to marine heatwaves fully ameliorated its lethal effect on tropical copepod Pseudiaptomus incisus with a cost of reduced reproductive success constrained by the lowered grazing. Fish predation stress played a minor role in shaping the copepod performance, both within and across generations with or without marine heatwaves.

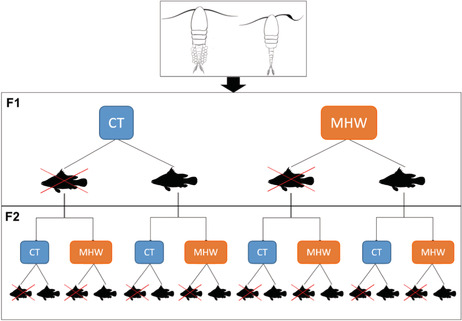

## 1 INTRODUCTION

Marine heatwaves (MHWs) are discrete periods of abnormally high temperatures (≥5 days) above the 90th percentile of 30‐year sea surface temperatures measured at the locality (Hobday et al., 2016; Oliver et al., 2018). In the last decade, MHWs have emerged as a major driver reshaping marine biodiversity, causing the tropicalization of temperate coastal ecosystems, mass coral bleaching, and mass mortality of coastal invertebrates worldwide (Garrabou et al., 2009, 2019; Hughes et al., 2017; Smale et al., 2019). The effects of MHWs on marine ecosystems and biota are becoming more severe under ongoing climate change (Smale et al., 2019). In the Southeast Asian region, coastal marine organisms are increasingly being exposed to episodes of MHWs (Feng et al., 2022; Yao et al., 2020). The average duration of MHWs in the Southeast Asian seas is 18–22 days per event (Yao & Wang, 2021), which is equivalent to ~2–3 generations of tropical coastal invertebrates. A few recent studies have found strong negative effects of MHWs on marine organisms such as copepods and fish in the Southeast Asian region (e.g., Doan et al., 2019; Le et al., 2020; Nguyen, Le, Doan, Pham, et al., 2020), but these were mostly limited to one‐exposed generation. It remains to be explored how transgenerational plasticity to MHWs might ameliorate the effects of warming on offspring generation (see, e.g., in coral fish, Donelson et al., 2012).

Advancements in eco‐evolutionary studies on adaptations of organisms to toxic algae, warming, ocean acidification, and contaminants have further explored the critical role of transgenerational plasticity (TGP) where the environment experienced by the parental generation may improve offspring performance in the same environment (Colin & Dam, 2005; Donelson et al., 2012; Donelson et al., 2018; Fox et al., 2019; Guillaume et al., 2016; Krause et al., 2017; Munday, 2014; Thor & Dupont, 2015). For example, the tropical coral damselfish *A. polyacanthus* reduced the aerobic scope by 15–30% under acute exposure to an elevated temperature of +1.5°C and + 3°C, but the offspring's aerobic scope fully recovered when reared at the same temperature (Donelson et al., 2012). TGP is especially crucial for organisms to cope with new, predictable but fast‐changing and short‐term environmental changes across generations (Donelson et al., 2018). The duration of an MHW often lasts longer than one generation for nearly all tropical zooplankton species. However, the duration of an MHW is often not long enough for directional evolutionary adaptation, as occurs in response to seasonal changes in water temperature (Sasaki & Dam, 2020) or long‐term warming (Dam et al., 2021; Dinh Van et al., 2013). TGP generally occurs through epigenetic changes, habitat selection, or niche construction (reviewed in Donelan et al., 2020). In particular, epigenetic changes such as the methylation of genes encoding oxygen consumption, mitochondrial activity, and energy homeostasis play crucial functions in restoring the performance of stress‐exposed organisms across generations (Ryu et al., 2018). Besides TGP, the parental effect is another non‐genetic transgenerational effect, as the transfer of nutrients from mothers to eggs may also affect offspring performance (reviewed in Ho & Burggren, 2010). Parental effects of warming may negatively affect offspring performance, which has been shown in mosquito larvae with increased mortality and delayed development (Tran et al., 2018). Alternatively, genetic selection may occur through stressor‐induced mortality (Krause et al., 2017) or selection in favor of higher hatching success (Dam et al., 2021), which removes the most sensitive genotypes or increases the tolerant genotypes in the population, and may contribute to increased fitness of the offspring generation.

In the shallow tropical coastal ecosystems such as seagrasses, mangroves, and coral reefs, the predation stress is typically high as these ecosystems are the spawning and nursery ground of marine species. Non‐consumptive predation stress from voracious fish larvae and juveniles can significantly influence prey morphology, behavior, physiology, growth, and reproduction (e.g., Bjærke et al., 2014; Lasley‐Rasher & Yen, 2012; Truong et al., 2020). For example, the escape ability of copepods *Notodiaptomus conifer* and *Argyrodiaptomus falcifer* can be enhanced (Gutiérrez et al., 2010). Calanoid copepods commonly show an increase in growth (e.g., *Temora longicornis*, Bjærke et al., 2014) and reproduction (e.g., *P. incisus*, Truong et al., 2020) as general life‐history trait responses to fish kairomones. Parental exposure to predators may also induce an increase in the reproduction of offspring generation (e.g., *Daphnia magna*) and this effect may last two generations after exposure to predators (Walsh et al., 2015). However, the TGP of prey species to predation stress may reduce in the degree of plasticity with an increasing number of exposed generations exposed to predators. For example, the pea aphid (*Acyrthosiphon pisum*) responds to the presence of the predator ladybirds (*Harmonia axyridis*) by producing a higher frequency of the winged offspring, but this TGP response decreases across 22 exposed generations (Sentis et al., 2018).

Investigations of the transgenerational effect of MHWs in an ecologically relevant context, such as the presence of fish predator cues (FPC) on key zooplankton species, are relevant and timely with the increasing frequency, severity, and duration of MHWs and the intense predation stress of tropical coastal environments. Understanding whether copepods are resilient or vulnerable to MHWs in the context of predation stress is important, given that they are a key pathway for the transfer of energy and resources from photosynthesizing organisms to higher trophic levels, and ultimately the productivity of the coastal ecosystems (Chew et al., 2012). However, the combined effect of heatwaves and non‐consumptive predation risk on prey species across generations is still a major knowledge gap in current ecological research. Our previous study shows that FPC induced a higher individual performance of the calanoid copepod *Pseudodiaptomus incisus* under control temperature, but it magnified the deleterious impacts of MHW on grazing and reproductive success (Truong et al., 2020). In this study, we address the knowledge gap identified above by assessing the immediate effect during the exposure (Dinh et al., 2016) together with the effects of parental exposure, TGP (parental and offspring exposure) to MHW, FPC, and their interactions in a full orthogonal manner with 4 treatments in F1 and 16 treatments in F2 generation. We tested the susceptibility of *P. incisus* to MHW and FPC by following eight hypotheses:

MHW reduces the performance of *P. incisus* due to energetic constraints under extreme warming (Truong et al., 2020).

FPC increases the performance of *P. incisus* as a general antipredator response (Bjærke et al., 2014; Truong et al., 2020).

FPC‐induced increase in performance of *P. incisus* is not sustained under MHW due to the energetic constraints (Truong et al., 2020).

Parental exposure to MHW reduces offspring performance in the control temperature due to poor maternal provisioning (Tran et al., 2018).

Parental exposure to FPC increases offspring performance in the absence of FPC (Walsh et al., 2015).

TGP to MHW ameliorates the MHW effect on *P. incisus* offspring (Donelson et al., 2012).

TGP to FPC decreases the *P. incisus* offspring performance toward antipredator responses (Sentis et al., 2018).

The magnitude of TGP to MHW is altered by TGP to FPC, and *vice versa*. This is predicted based on the different types of responses of *P. incisus* to TGP to MHW and FPC.

## 2 MATERIALS AND METHODS

### 2.1 The tropical calanoid *Pseudodiaptomus incisus*

*Pseudodiaptomus incisus* is a calanoid copepod that can be found abundantly in aquaculture ponds, bays, lagoons, mangroves, and estuaries in Vietnam (BISMAL, 2020; Truong et al., 2014). They develop from the first nauplii to adults in 8–14 days, depending on temperatures (25–35°C) and salinity (5–40 PSU) (Nguyen, Le, Doan, Nguyen, et al., 2020). At temperatures of 30–34°C, the adult lifespan of *P. incisus* varies from 11 to 24 days (Truong et al., 2020).

We used a zooplankton net (mesh size = 200 μm) to collect *Pseudodiaptomus incisus* from a coastal aquaculture pond (11.82397°N, 109.1233°E) in Cam Ranh Bay, July 2020. There were no barramundi in the pond. Copepods were transferred to the Copepod Laboratory at Cam Ranh Centre for Tropical Marine Research and Aquaculture, Nha Trang University. The pond water salinity and temperature were 27–28°C and 35 PSU, respectively. Healthy adult copepods (F0) were sorted and then divided into 5‐L bottles, approximately 1200 individuals per bottle, for acclimation. Adult males and females were acclimated in water baths at 30°C or 34°C for 3 days. The temperature was increased by 1°C every 12 h until reaching the experimental temperatures. During the acclimation, the salinity, light: dark cycle, and dissolved oxygen concentration were kept at 30 PSU, 12 L:12D, and >5 mg L^−1^ by aerations, respectively (see also Truong et al., 2020). *P. incicus* were fed two times a day with *Isochrysis galbana* at 30,000–33,000 cell L^−1^ (~800–850 μg carbon per litter, Doan et al., 2018).

### 2.2 Fish predator cue preparation

Barramundi larvae *Lates calcarifer* (15 individuals with a total length of 14 ± 1 mm) were reared in a 1‐L bottle. Fish larvae were fed with ~100 *P. incisus* twice a day. After three rearing days, barramundi larvae were removed and the rearing water containing fish predator cues (FPC) was filtered through a 0.5 μm filter paper. This filtered water with FPC was divided into 50 ml aliquots and subsequently frozen at −20°C. FPC was thawed before being used in the experiment, as predator cue effects on the prey still remain after being frozen (Lürling & Scheffer, 2007).

### 2.3 Transgenerational experiment

**FIGURE 1:**
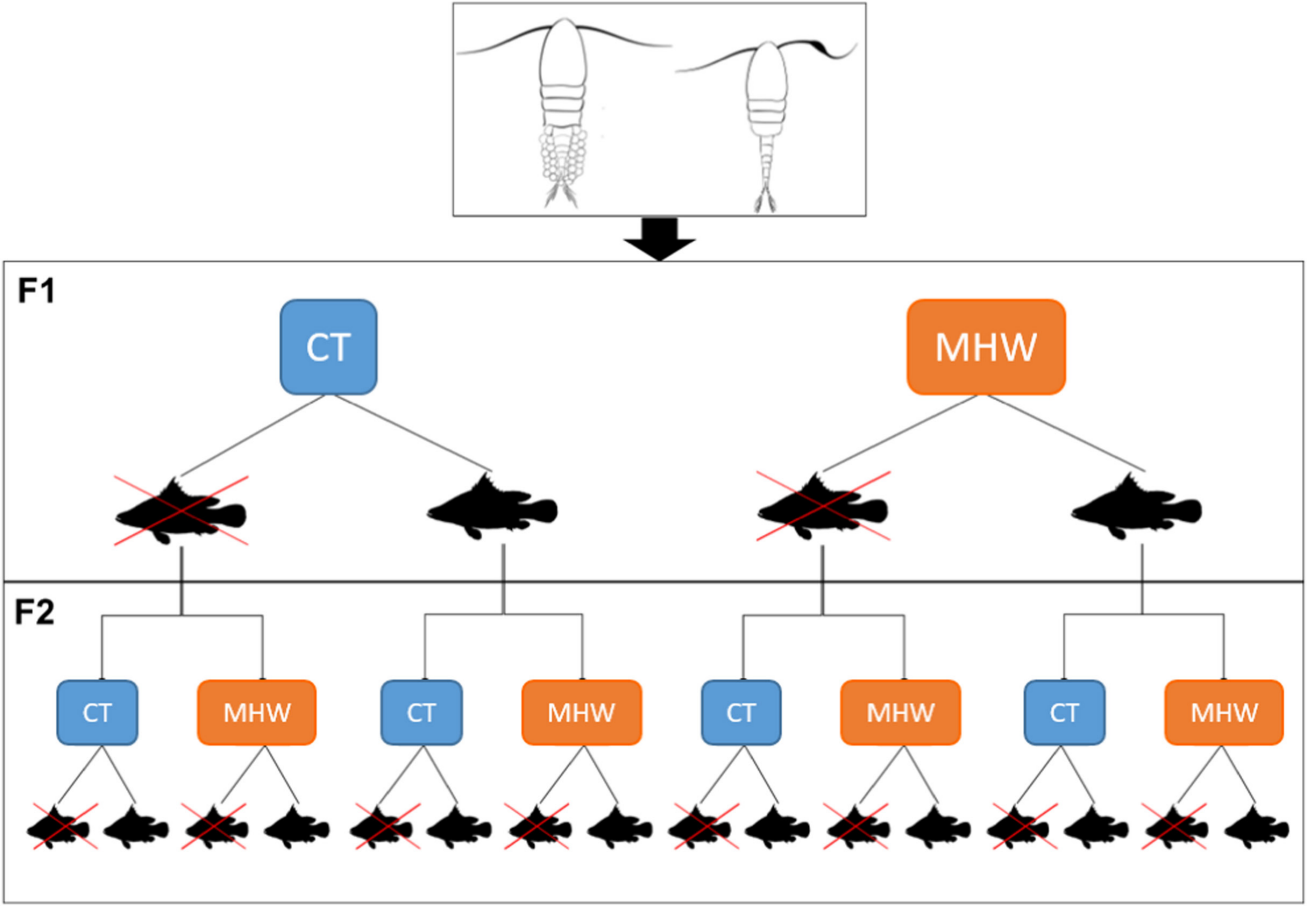
The schematic overview of the transgenerational experiment for the immediate and transgenerational MHW and FPC effects on *Pseudodiaptomus incisus* (CT = control temperature).

To test the transgenerational effect of MHW and FPC on the performance of the tropical copepod *Pseudodiaptomus incisus*, effects on two generations of *P. incisus* were studied (F1 and F2). F1 *P. incisus* were exposed to one of four treatments, including two thermal treatments (30 or 34°C) × 2 FPC (presence or absence) × 10 replicates (Figure 1). The control temperature of 30°C was chosen as it is the mean coastal water temperature in the coastal water in southern Vietnam (see Appendix S1, Doan et al., 2018). We manipulated an experimental MHW condition with a temperature of 34°C, which is about ~2°C higher than the 90% temperature variations measured in the Cam Ranh Bay (Doan X.N., Pham, Q.H. and Dinh K.V., unpublished data).

To start the experiment, acclimatized F0 *P. incisus* carrying egg sacs (prosomal length = 797.43 ± 2.17 μm, clutch size = 16 ± 2 eggs) were assigned to 1.2‐L plastic bottles (15 females each bottle) and fed with *I. galbana* for acclimation. After 30 h, 180–240 F1 nauplii were assigned to each experimental bottle; those bottles were pre‐acclimated to experimental temperatures. FPC solution or filtered seawater (1 ml) was added to each experimental bottle. The rearing medium and FPC and algae were renewed daily to minimize the change in the FPC concentration and the indirect effect of MHW on the algal quality (Truong et al., 2020).

Once F1 developed into adults, 20 F1 females carrying egg sacs in each of 10 bottles of each treatment (200 individuals per F1 treatment) were collected and transferred into 20 bottles (10 F1 females per bottle) and incubated for 30 h. F2 offspring in every F1 treatment were divided into four groups, corresponding to four experimental conditions 30°C – no FPC, 30°C – FPC, 34°C – no FPC, and 34°C – FPC, resulting in 16 treatments (Figure 1).

In both generations, we analyzed clutch size (from fixed females carrying an egg sac), percentage of females with hatched eggs, and the number of hatched nauplii per clutch from alive females. Ten other males and females were used to test for survival of males and females, cumulative nauplii, and fecal pellets over 5 days. All data collection procedure was similar to our previous study (Truong et al., 2020).

### 2.4 Cumulative nauplii and fecal pellets

To evaluate the reproductive output and the grazing rate of *P. incisus*, we quantified the cumulative nauplii and fecal pellets in 5 days. Ten adults of both sexes were transferred to a separated 1‐L bottle (10 replicates per treatment). Bottles were daily filtered using the same filtering net (see above) to collect the nauplii and fecal pellets. Alive adults were returned to the bottle while the dead ones were removed. The content containing nauplii and fecal pellets was transferred to a Petri dish and fixed with Lugol (4%). The number of nauplii and fecal pellets was counted using a stereomicroscope (SZ51, Olympus, Japan). In calanoid copepods, the fecal pellets were used as the index of the ingestion (Besiktepe & Dam, 2002), which typically has a positive correlation with egg production (Besiktepe & Dam, 2020).

### 2.5 Statistical analyses

The data were analyzed using the R program (version 4.1.3 released on 2022‐03‐10). MHW and FPC (F1 generation) and F1‐MHW, F1‐FPC, F2‐MHW, and F2‐FPC (F2 generation) were fixed covariates in the statistical models. Survival of females and males, as the response variable, was defined as continuous proportions ranging from 0 to 1 as quasibinomial distribution using the generalized linear models (GLM) with the F‐test. Hatching success was incorporated as a binomial distribution in the GLM with the Chi‐square test. A Poisson GLM with a log‐link function was used to model the clutch size and hatched nauplii per clutch as a function of the fixed covariates. We modeled cumulative nauplii per female and cumulative fecal pellets per individual, which were average values of the observations on each replicate, as a quasi‐Poisson distribution using GLM with *F*‐test. The interaction terms in the models were MHW × FPC (F1 generation) and the two‐way, three‐way, and four‐way interactions of F1‐MHW, F1‐FPC, F2‐MHW, and F2‐FPC (F2 generation).

The fitted models of binomial and Poisson distributions were checked for overdispersion. We refitted the models having overdispersion by applying the following solutions: (i) removing outliers of the response variables, (ii) defining the response variables as *quasi‐*distribution in the models, (iii) including random factors using generalized linear mixed‐effects models (GLMER, package *lme4* version 1.1–29), and/or (iv) changing to a negative binomial model using negative binomial generalized linear model (GLM.NB, package *MASS* version 7.3–57). The possible models were then validated using (i) Akaike information criterion (AIC) if available, (ii) plotting and assessing residuals versus fitted values, (iii) evaluating predicted versus actual values of response variables, and (iv) checking the normal distribution of the residuals. The best‐fit model is the one that has the smallest AIC, minor errors in the predicted values, and more normally distributed residuals.

## 3 RESULTS

### 3.1 F1 generation

**FIGURE 2:**
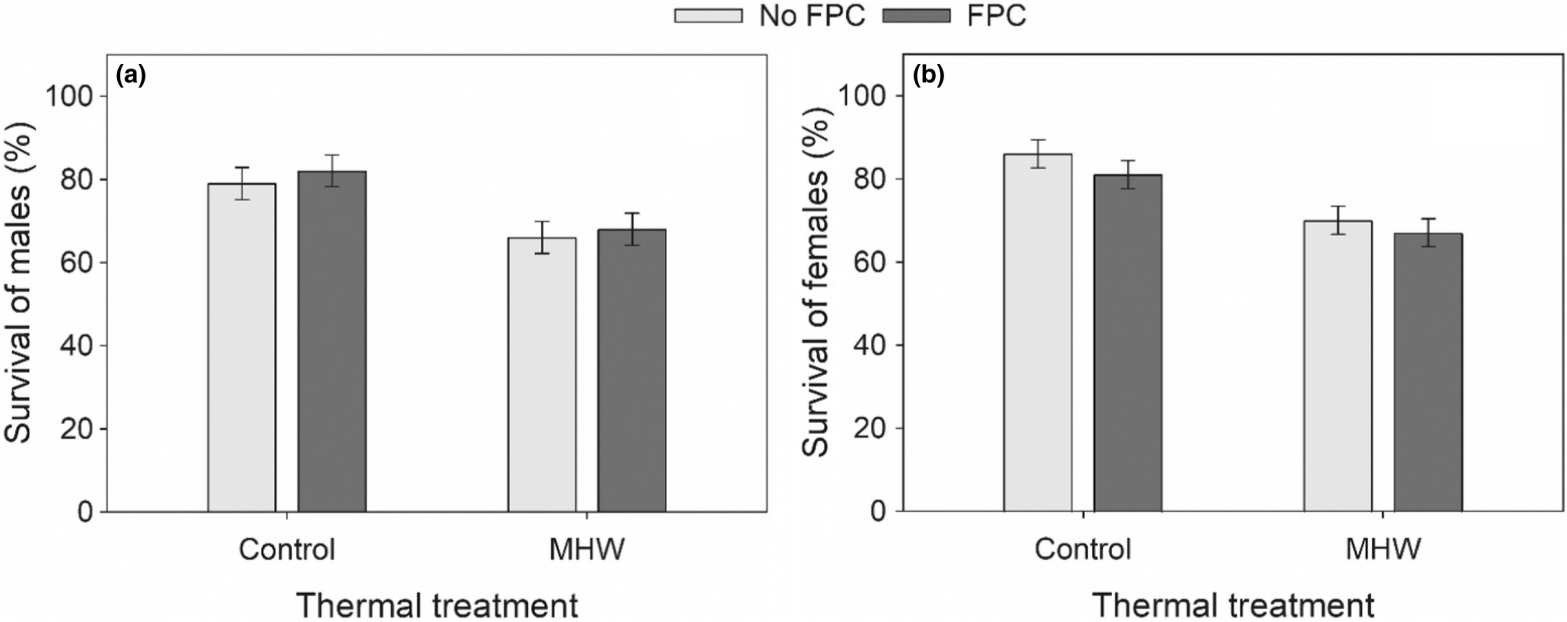
Effects of the marine heatwave (MHW) and fish predator cues (FPC) on the survival (mean ± SE) of males (a) and females (b) in F1 *Pseudodiaptomus incisus* males (a) and females (b).

Exposure to MHW reduced the survival of both males and females by 17%–18% (MHW, Table 1, Figure 2, in agreement with H1). The lethal effect of MHW was independent of FPC exposure, indicated by an insignificant MHW × FPC interaction (Table 1, Figure 2, in contrast with H3).

**TABLE 1:** The results of the statistical analyses testing effects of marine heatwave (MHW) and fish predator cues (FPC) on survival, reproductive parameters, and cumulative fecal pellets of F1 *Pseudodiaptomus incisus*. Significant *p* values are signed with *.

Response variables	Models	Factors	*df*1	*df*2	*F*	*p*
Female survival	GLM – quasibinomial	MHW	1	38	33.80	<.001*
		FPC	1	37	2.45	.126
		MHW × FPC	1	36	0.58	.453
Male survival	GLM – quasibinomial	MHW	1	38	24.96	<.001*
		FPC	1	37	0.87	.357
		MHW × FPC	1	36	0.12	.728
Clutch size	GLM – Poisson	MHW	1	398	NA	<.001*
		FPC	1	397	NA	.001*
		MHW × FPC	1	396	NA	.976
% females with hatched eggs	GLM – binomial	MHW	1	478	NA	<.001*
		FPC	1	477	NA	.667
		MHW × FPC	1	476	NA	>.99
Hatched nauplii clutch‐1	GLM.NB – negative binomial	MHW	NA	NA	NA	<.001*
		FPC	NA	NA	NA	<.001*
		MHW × FPC	NA	NA	NA	.364
Cumulative nauplii female^−1^	GLM – quasi‐Poisson	MHW	1	38	1789.84	<.001*
		FPC	1	37	78.25	<.001*
		MHW × FPC	1	36	55.75	<.001*
Cumulative fecal pellets ind^−1^	GLM – quasi‐Poisson	MHW	1	38	2641.95	<.001*
		FPC	1	37	792.01	<.001*
		MHW × FPC	1	36	19.86	<.001*

**FIGURE 3:**
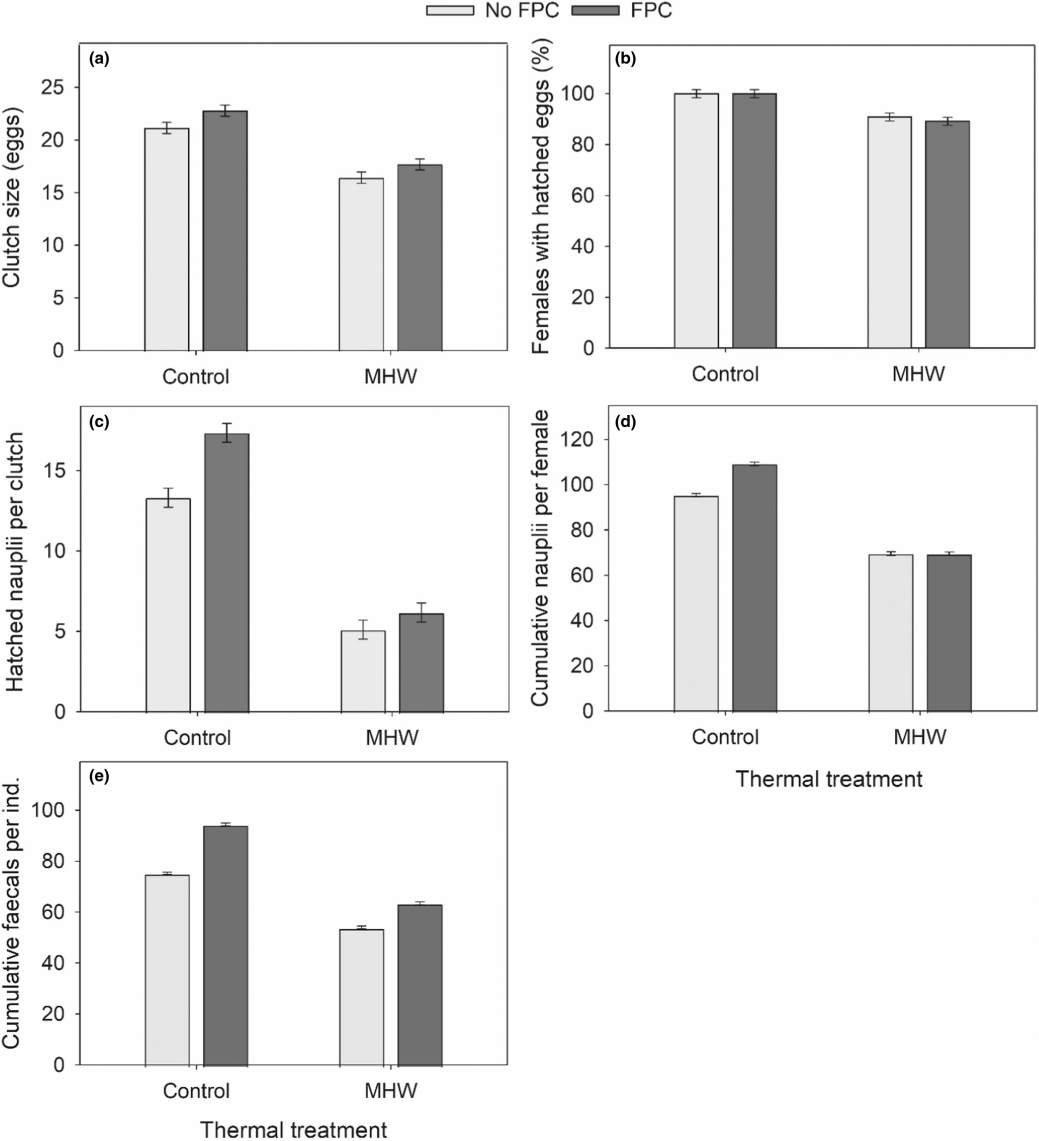
Effects of the marine heatwave (MHW) and fish predator cues (FPC) on the number of eggs per clutch (a), % females produced hatched eggs (b), hatched nauplii hatched from a clutch (c), cumulative nauplii per female (d), and fecal pellets per individual (e) of F1 *Pseudodiaptomus incisus*. Data are visualized as mean ± SEs.

The reproductive success and grazing of *P. incisus* were overall affected negatively by MHW (MHW, Table 1, Figure 3a–e, in agreement with H1). The clutch size, the percentage of females with hatched eggs, and hatched nauplii per clutch dropped by 22, 9, and 62%, respectively, at 34°C (Table 1, Figure 3c). The cumulative nauplii and fecal pellets were 27% and 28% lower in MHW than in the control temperature (Table 1, Figure 3d,e). The FPC effect on the percentage of females with hatched eggs was insignificant (Table 1, Figure 3b, in contrast with H2). For the clutch size, the effect of FPC was statistically insignificant in both temperatures (MHW × FPC, Table 1, Figure 3a, in contrast with H3). The number of hatched nauplii per clutch and cumulative fecal pellets of *P. incicus* increased in the presence of FPC (in agreement with H2), but the FPC effect was several times higher in the control temperature than in MHW (MHW × FPC, Table 1, Figure 3c,e, in agreement with H3). *P. incisus* produced approximately 15% more cumulative nauplii in the presence of FPC only in control temperature, but not in MHW (MHW × FPC, Table 1, Figure 3d, agreement with H1 and H3). The correlation of cumulative nauplii production and cumulative fecal pellets was insignificant (*F*_1,36_ = 2.65, *p* = .11, slope ± 1 *SE* = 0.38 ± 0.24, *R* ^2^ = 0.89).

### 3.2 F2 generation

**FIGURE 4:**
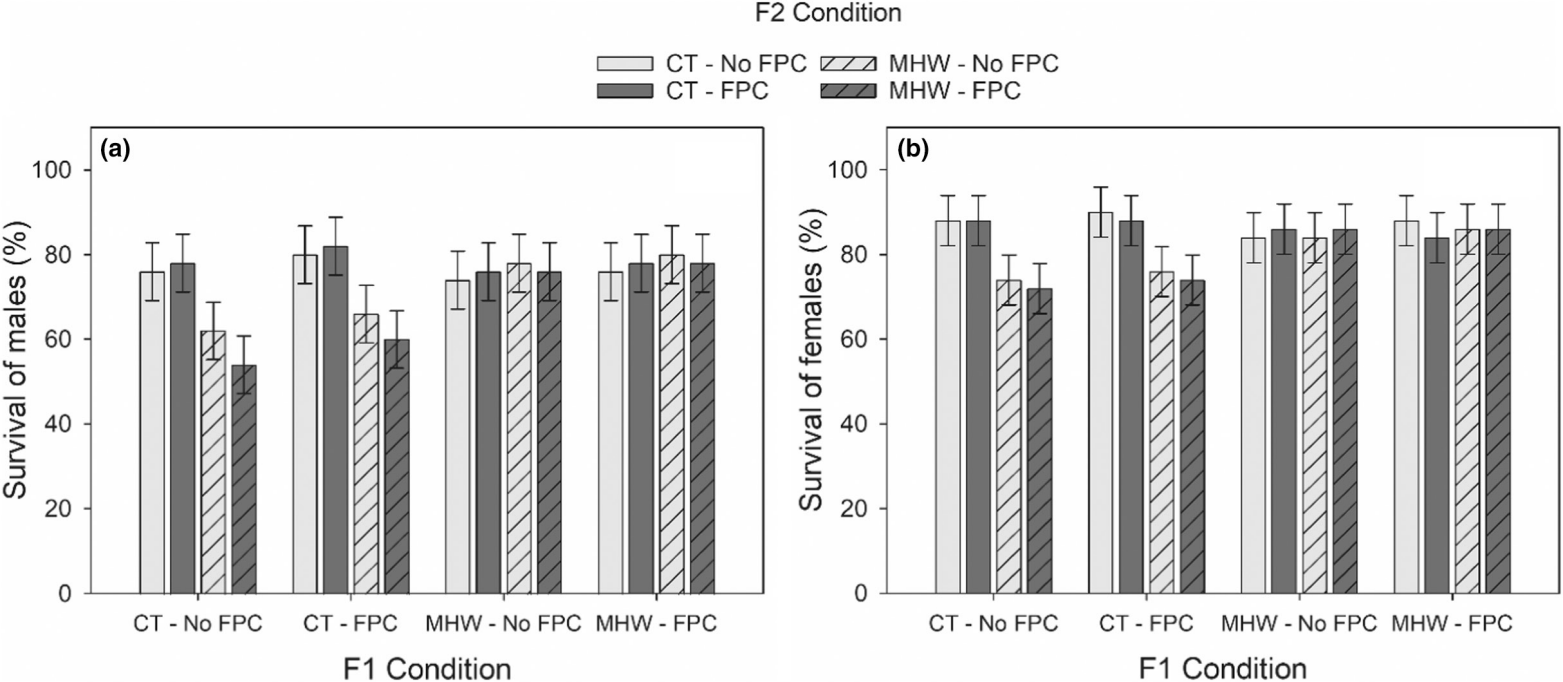
Immediate and transgenerational effects of the marine heatwave (MHW) and fish predator cues (FPC) on the survival (mean ± *SE*) of F2 *Pseudodiaptomus incisus* males (a) and females (b). *CT: Control temperature.

Survival of F2 *P. incisus* females and males was reduced by 8%–10% under MHWs (F2‐MHW, Table 2, Figure 4, in agreement with H1). FPC did not affect F2 female and male survival as the F2‐FPC effect was insignificant (Table 2, Figure 4, in contrast with H2). F2‐FPC did not alter the lethal effect of F2‐MHW, as indicated by an insignificant interaction of F2‐MHW × F2‐FPC (Table 2, Figure 4, in contrast to H3). Strikingly, TGP to MHW (F1‐MHW × F2‐MHW) resulted in a similar female and male survival compared to the control temperature (F1‐MHW × F2‐MHW, Table 2, Figure 4, in agreement with H6), suggesting that lethal F2‐MHW effect was ameliorated. This pattern was independent of F1‐FPC and F2‐FPC (Table 2, Figure 4, in contrast with H8). The lethal MHW effect in the parental generation was no longer present in F2 generation when F2 copepods were reared in the control temperature (F1‐MHW, Table 2, Figure 4, in contrast with H5). There were no lethal effects of PFC on the survival of F2 males and females and it was independent of F1‐FPC (in contrast with H7).

**TABLE 2:** The results of statistical analyses testing the immediate and transgenerational effects of marine heatwave (MHW) and fish predator cues (FPC) on survival, reproductive parameters, and cumulative fecal pellets of F2 *Pseudodiaptomus incisus*. Significant *p* values are signed with *.

Response variables	Models	Factors	*df*1	*df*2	*F*	*p*
Female survival	GLM – quasibinomial	F1‐MHW	1	78	7.62	.008*
		F1‐FPC	1	77	0.66	.42
		F2‐MHW	1	76	22.36	<.001*
		F2‐FPC	1	75	0.24	.626
		F1‐MHW × F1‐FPC	1	74	0.01	.936
		F1‐MHW × F2‐MHW	1	73	18.81	<.001*
		F1‐FPC × F2‐MHW	1	72	0.00	.99
		F1‐MHW × F2‐FPC	1	71	0.20	.653
		F1‐FPC × F2‐FPC	1	70	0.70	.406
		F2‐MHW × F2‐FPC	1	69	0.11	.744
		F1‐MHW × F1‐FPC × F2‐MHW	1	68	0.00	.992
		F1‐MHW × F1‐FPC × F2‐FPC	1	67	0.30	.584
		F1‐MHW × F2‐MHW × F2‐FPC	1	66	0.13	.719
		F1‐FPC × F2‐MHW × F2‐FPC	1	65	0.33	.57
		F1‐MHW × F1‐FPC × F2‐MHW × F2‐FPC	1	64	0.02	.885
Male survival	GLM – quasibinomial	F1‐MHW	1	78	17.87	<.001*
		F1‐FPC	1	77	3.61	.062
		F2‐MHW	1	76	23.34	<.001*
		F2‐FPC	1	75	0.54	.466
		F1‐MHW × F1‐FPC	1	74	0.33	.57
		F1‐MHW × F2‐MHW	1	73	31.93	<.001*
		F1‐FPC × F2‐MHW	1	72	0.01	.94
		F1‐MHW × F2‐FPC	1	71	0.48	.491
		F1‐FPC × F2‐FPC	1	70	0.01	.907
		F2‐MHW × F2‐FPC	1	69	3.27	.075
		F1‐MHW × F1‐FPC × F2‐MHW	1	68	0.01	.917
		F1‐MHW × F1‐FPC × F2‐FPC	1	67	0.02	0.895
		F1‐MHW × F2‐MHW × F2‐FPC	1	66	0.28	.601
		F1‐FPC × F2‐MHW × F2‐FPC	1	65	0.00	.952
		F1‐MHW × F1‐FPC × F2‐MHW × F2‐FPC	1	64	0.01	.925
Clutch size	GLM – quasi‐Poisson	F1‐MHW	1	798	41.43	<.001*
		F1‐FPC	1	797	4.40	.036*
		F2‐MHW	1	796	630.33	<.001*
		F2‐FPC	1	795	14.05	<.001*
		F1‐MHW × F1‐FPC	1	794	5.49	.019*
		F1‐MHW × F2‐MHW	1	793	248.12	<.001*
		F1‐FPC × F2‐MHW	1	792	4.96	.026*
		F1‐MHW × F2‐FPC	1	791	3.88	.049*
		F1‐FPC × F2‐FPC	1	790	6.98	.008*
		F2‐MHW × F2‐FPC	1	789	1.05	.307
		F1‐MHW × F1‐FPC × F2‐MHW	1	788	20.89	<.001*
		F1‐MHW × F1‐FPC × F2‐FPC	1	787	12.30	<.001*
		F1‐MHW × F2‐MHW × F2‐FPC	1	786	13.90	<.001*
		F1‐FPC × F2‐MHW × F2‐FPC	1	785	4.76	.030*
		F1‐MHW × F1‐FPC × F2‐MHW × F2‐FPC	1	784	19.91	<.001*
% females with hatched eggs	GLM – binomial	F1‐MHW	1	958	NA	.003*
		F1‐FPC	1	957	NA	.718
		F2‐MHW	1	956	NA	<.001*
		F2‐FPC	1	955	NA	.463
		F1‐MHW × F1‐FPC	1	954	NA	.535
		F1‐MHW × F2‐MHW	1	953	NA	.002*
		F1‐FPC × F2‐MHW	1	952	NA	.851
		F1‐MHW × F2‐FPC	1	951	NA	.693
		F1‐FPC × F2‐FPC	1	950	NA	.301
		F2‐MHW × F2‐FPC	1	949	NA	.876
		F1‐MHW × F1‐FPC × F2‐MHW	1	948	NA	1
		F1‐MHW × F1‐FPC × F2‐FPC	1	947	NA	.002*
		F1‐MHW × F2‐MHW × F2‐FPC	1	946	NA	1
		F1‐FPC × F2‐MHW × F2‐FPC	1	945	NA	1
		F1‐MHW × F1‐FPC × F2‐MHW × F2‐FPC	1	944	NA	1
Hatched nauplii clutch^−1^	GLM.NB –negative binomial	F1‐MHW	NA	NA	NA	<.001*
		F1‐FPC	NA	NA	NA	.067
		F2‐MHW	NA	NA	NA	<.001*
		F2‐FPC	NA	NA	NA	.246
		F1‐MHW × F1‐FPC	NA	NA	NA	.255
		F1‐MHW × F2‐MHW	NA	NA	NA	<.001*
		F1‐FPC × F2‐MHW	NA	NA	NA	.316
		F1‐MHW × F2‐FPC	NA	NA	NA	.454
		F1‐FPC × F2‐FPC	NA	NA	NA	.078
		F2‐MHW × F2‐FPC	NA	NA	NA	.256
		F1‐MHW × F1‐FPC × F2‐MHW	NA	NA	NA	.161
		F1‐MHW × F1‐FPC × F2‐FPC	NA	NA	NA	.312
		F1‐MHW × F2‐MHW × F2‐FPC	NA	NA	NA	.155
		F1‐FPC × F2‐MHW × F2‐FPC	NA	NA	NA	.295
		F1‐MHW × F1‐FPC × F2‐MHW × F2‐FPC	NA	NA	NA	.141
Cumulative nauplii female^−1^	GLM – quasi‐Poisson	F1‐MHW	1	78	174.73	<.001*
		F1‐FPC	1	77	5.21	.026*
		F2‐MHW	1	76	1170.72	<.001*
		F2‐FPC	1	75	11.05	.001*
		F1‐MHW × F1‐FPC	1	74	28.90	<.001*
		F1‐MHW × F2‐MHW	1	73	937.27	<.001*
		F1‐FPC × F2‐MHW	1	72	13.64	<.001*
		F1‐MHW × F2‐FPC	1	71	1.82	.183
		F1‐FPC × F2‐FPC	1	70	12.72	<.001*
		F2‐MHW × F2‐FPC	1	69	4.93	.030*
		F1‐MHW × F1‐FPC × F2‐MHW	1	68	10.05	.002*
		F1‐MHW × F1‐FPC × F2‐FPC	1	67	0.12	.729
		F1‐MHW × F2‐MHW × F2‐FPC	1	66	0.00	.971
		F1‐FPC × F2‐MHW × F2‐FPC	1	65	3.23	.077
		F1‐MHW × F1‐FPC × F2‐MHW × F2‐FPC	1	64	0.13	.717
Cumulative fecal pellets ind^−1^	GLM – quasi‐Poisson	F1‐MHW	1	78	27.57	<.001*
		F1‐FPC	1	77	14.04	<.001*
		F2‐MHW	1	76	964.80	<.001*
		F2‐FPC	1	75	4.62	.035*
		F1‐MHW × F1‐FPC	1	74	22.84	<.001*
		F1‐MHW × F2‐MHW	1	73	724.00	<.001*
		F1‐FPC × F2‐MHW	1	72	41.82	<.001*
		F1‐MHW × F2‐FPC	1	71	1.06	.307
		F1‐FPC × F2‐FPC	1	70	11.12	.001*
		F2‐MHW × F2‐FPC	1	69	5.89	.018*
		F1‐MHW × F1‐FPC × F2‐MHW	1	68	0.16	.688
		F1‐MHW × F1‐FPC × F2‐FPC	1	67	1.40	.241
		F1‐MHW × F2‐MHW × F2‐FPC	1	66	6.48	.013*
		F1‐FPC × F2‐MHW × F2‐FPC	1	65	5.59	.021*
		F1‐MHW × F1‐FPC × F2‐MHW × F2‐FPC	1	64	2.19	.144

**FIGURE 5:**
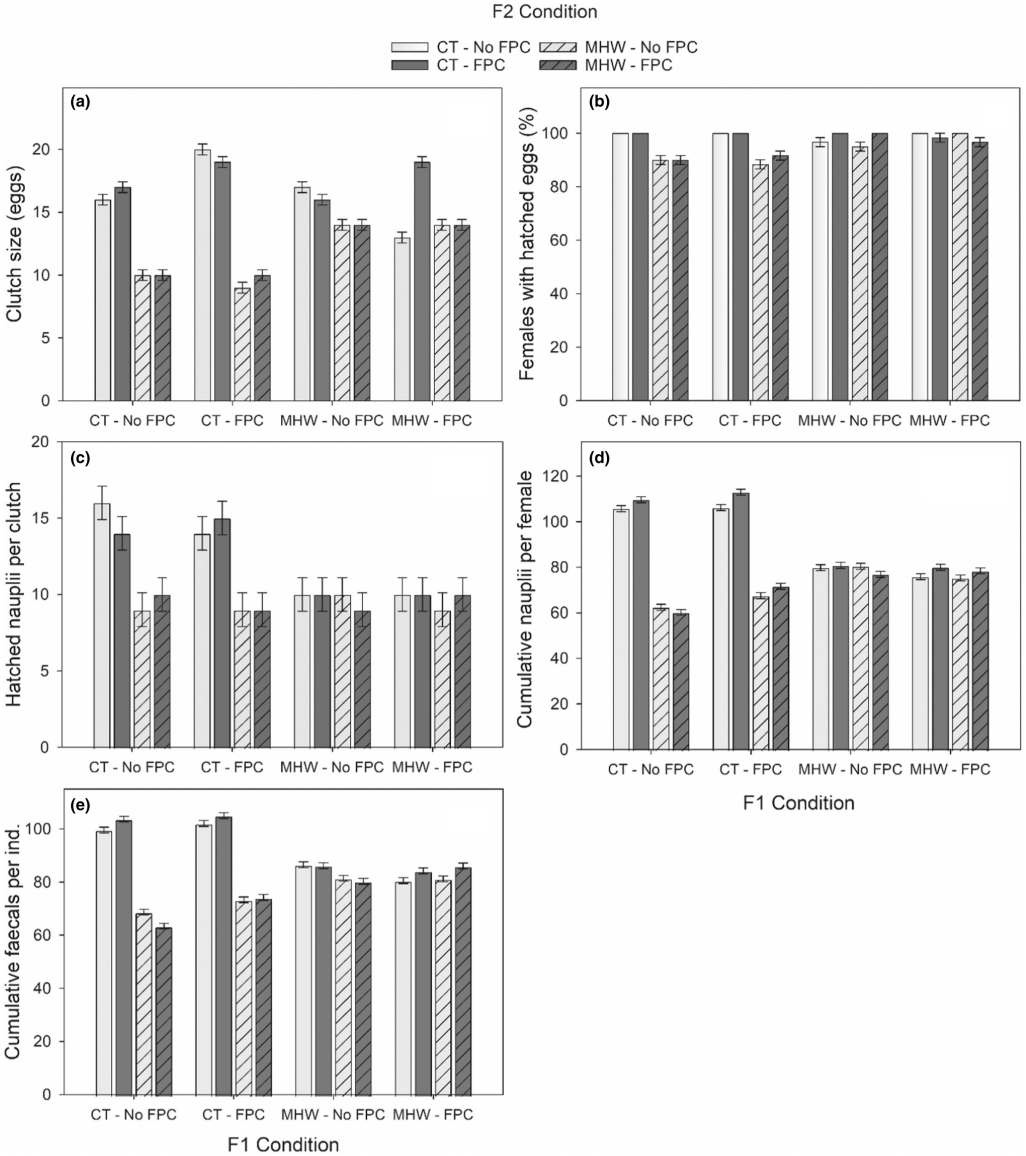
The number of eggs per clutch (a), % females produced hatched eggs (b), hatched nauplii egg clutch (c), cumulative nauplii per female (d), and fecal pellets per individual (e) of F2 *Pseudodiaptomus incisus*. Data are visualized as mean ± SEs. *CT: Control temperature.

The clutch size, % females with hatched eggs, and hatched nauplii from a clutch of F2 *P. incisus* were 42%, 5%, and 22% reduced in F2‐MHW (F2‐MHW, Table 2, Figure 5a–c, in agreement with H1), respectively. The F2‐MHW effect was independent of the F2‐FPC as F2‐MHW × F2‐FPC was insignificant (in contrast with H3). Importantly, F2‐MHW was generally less intense when F1 was also exposed to MHW (F1‐MHW × F2‐MHW, Table 2, Figure 5a–c, in partial agreement with H6). Parental exposure to MHW (F1‐MHW) also resulted in 22% and 33% reductions in the size of clutches and hatched nauplii from a clutch of F2 *P. incisus* (in agreement with H4). No F1‐MHW effect on % F2 females with hatched eggs occurred, as it was similar to the control level (F1‐MHW, Table 2, Figure 5a–c, in contrast with H4). Overall, F2‐FPC caused an increased clutch size of F2 females by 4% (in agreement with H2), but this pattern was mainly driven by large clutches of control F2 females whose parental generation was also exposed to both MHW and FPC (F1‐MHW × F1‐FPC), resulting in two‐, three‐, and four‐way interactions (Table 2, Figure 5a). The F2‐FPC effect on other reproductive parameters was absent or minimal (F2‐FPC, Table 2, Figure 5b–d, partly in agreement with H2).

Overall, cumulative nauplii and fecal pellets of F2 copepods decreased by 40 and 32% with MHW exposure, respectively (F2‐MHW, Table 2, Figure 5d,e, in agreement with H1). Parental exposure to MHW (F1‐MHW) also resulted in lower cumulative nauplii and fecal pellets than the control copepods (F1‐MHW, Figure 5b–e, in agreement with H4). F2‐MHW induced reductions in cumulative nauplii, and fecal pellets were ~12% less strong in F2 copepods whose parental generation was also exposed to MHW (F1‐MHW) (TGP F1‐MHW × F2‐MHW, Table 2, Figure 5d,e, in partial agreement with H6). The F2‐MHW effect was also 3%–4% less strong in F1‐FPC (F1‐FPC × F2‐MHW), but F1‐FPC did not influence the cumulative nauplii and fecal pellets of copepods that were exposed to MHW for both generations (F1‐MHW × F1‐FPC × F2‐MHW, Table 2, Figure 5d,e, in contrast with H8). F2‐FPC exposure caused a minor increase (~3%–4%) in both cumulative nauplii and fecal pellets of F2 *P. incisus*, but only at the control temperature and in F1‐FPC (F2‐MHW × F2‐FPC, F1‐FPC × F2‐FPC, in agreement with H3; F1‐FPC × F2‐MHW × F2‐FPC, Table 2, Figure 5e). F2‐FPC exposure caused a slightly (~3%) stronger reduction in cumulative fecal pellets in F2‐MHW‐exposed copepods, but only for those with no parental MHW exposure (F1‐MHW × F2‐MHW × F2‐FPC, Table 2, Figure 5e, in agreement with H3). Cumulative nauplii covaried positively with the cumulative fecal pellets (*F*_1,64_ = 30.3, *p* < .001, slope ± 1 *SE* = 0.68 ± 0.12, *R* ^2^ = .94).

## 4 DISCUSSION

This is the first study investigating the role of transgenerational effects in shaping the individual and interactive effects of marine heatwaves in combination with non‐consumptive predation stress on the tropical calanoid copepod *P. incisus*. We found a particularly strong effect of MHWs in the first generation, but the lethal MHW was fully ameliorated in the second generation. Interestingly, predation stress had little effect on the performance of *P. incisus* and played a minor role in shaping the transgenerational effects of MHWs on *P. incisus*. In the following paragraphs, we will first discuss the interactive effects of MHWs and non‐consumptive predation stress (using FPC from barramundi larvae), then the parental effect and transgenerational plasticity of both factors. Finally, we arrive at conclusions and applications for ecological risk assessment of MHWs on tropical zooplankton in the context of biotic interactions.

### 4.1 The effects of MHW (H1) and FPC (H2) and their interactions (H3)

Results in the F1 generation mostly confirmed and strengthened major findings of severe MHW impacts on *P. incisus* performance which were observed in our previous study focusing on the interactive effects of MHW and FPC on the life‐history traits of *P. incisus* for an entire single generation (Truong et al., 2020). Indeed, MHW exposure increased mortality, lowered hatching success, and cumulative nauplii and fecal pellets. Increased mortality likely occurred as a result of physiological dysfunctions, for example, the decline in ATP synthesis shows a tight correlation with copepod survival under thermal stress (Harada et al., 2019). Two other general, but not exclusive, physiological mechanisms for the warming‐induced mortality in ectotherms are the damage of macromolecules (Somero, 2010), and the higher cellular oxygen demand than the capacity of oxygen delivery (Pörtner, 2010). The reduced performance of *P. incisus* was particularly strong for reproductive success with ~30%–60% reductions in the size of egg clutches, the percentage of females producing hatched eggs, hatched nauplii from a clutch, and cumulative nauplii – all widespread phenomena in tropical copepods under exposure to MHW (Doan et al., 2019; Nguyen, Le, Doan, Pham, et al., 2020). The lower reproductive outputs are generally related to reduced grazing (Besiktepe & Dam, 2020; Krause et al., 2017), thereby energy intake. While we observed a lower cumulative fecal pellets of MHW‐exposed F1 *P. incisus*, the correlation of cumulative fecal pellets and nauplii was insignificant, suggesting that higher energy available for reproduction may be reduced due to higher energetic cost for basal maintenance under MHWs (Siegle et al., 2022).

The FPC presence caused a small increase in females with successfully hatched eggs, the number of nauplii hatched per clutch of *P. incisus*, which may be an adaptive response to predators. For example, predation can cause 50%–75% mortality in marine copepods, and increased reproductive outputs are a general mechanism to compensate for consumptive mortality (Hirst & Kiorboe, 2002). Importantly, the FPC‐induced increase in nauplii production only occurred at the control temperature but not under MHW exposure which suggests that copepods could not maintain high reproduction due to energetic cost (Low et al., 2018). Stronger MHW‐induced reductions in cumulative nauplii and fecal pellets were observed in FPC‐exposed *P. incisus* in our previous study (Truong et al., 2020), in which we could quantify both parameters for an entire adult lifespan.

### 4.2 Parental effects of MHW (H4) and FPC (H5) on the F2 generation

The parental effects of MHWs and/or FPC occurred when *P. incisus* was only exposed to these stressors in the F1 generation but not in the F2 generation. One of our important findings was that parental exposure to MHWs (F1‐MHW × F2‐control) reduced reproductive success and grazing, but not survival of F2 *P. incisus*. The reduced F2 reproductive success and cumulative fecal pellets may result from reduced energy and resource investments for F1 reproduction, further limited by the lower F1 grazing. F1‐MHW‐induced poor maternal provisioning was likely the reason for the lower performance of F2 copepods. A similar result has been observed in a previous study on the same copepod species (Dinh et al., 2021).

Across treatments, the main parental effect of FPC accounted for only a ~2% positive effect on the cumulative nauplii and fecal pellets. The positive effect of parental exposure to FPC on the reproductive outputs of offspring is common in zooplankton and this effect may last for several generations after removing the predation stress (see, e.g., in *Daphnia ambigua*, Walsh et al., 2015). However, these positive parental FPC effects on *P. incicus* were at least an order of magnitude smaller than the negative parental MHW effects on these life‐history traits and were unlikely to affect the overall sensitivity of *P. incicus* to MHW.

### 4.3 Transgenerational plasticity of P. incisus to MHW (H6), FPC (H7), and their interaction (H8)

Transgenerational plasticity of *P. incisus* to MHW and/or PFC occurred when copepods were exposed to these stressors for both generations F1 and F2. There is mixed evidence of transgenerational effects of multiple stressors on the vulnerability of aquatic species. On the one hand, there is evidence that corals (Torda et al., 2017) and coral fishes (Donelson et al., 2012) show rapid transgenerational acclimation to warming, and that can be linked to a complete compensation in aerobic scope (Donelson et al., 2012), the epigenetic changes (Ryu et al., 2018), or the associated microbes (Torda et al., 2017). On the other hand, there is evidence that transgenerational exposure to extreme warming, metal, fish predator cues (Pham et al., 2020), and pesticides (Tran et al., 2018) may reduce the offspring performance in same stressors as the cumulative impacts of stressors across generations. This study found the different degrees of TGP of *P. incisus* to MHWs in shaping F2 performance. Indeed, *P. incisus* showed rapid and complete TGP to the lethal MHW effect, as indicated by increased survival in F1‐MHW × F2‐MHW exposed males and females to the control temperature. Interestingly, FPC played a minor role in TGP to MHW as indicated by no or minor variation in the TGP effect size of MHW in the presence or absence of FPC, suggesting a dominant effect of MHW. More general, the TGP effect size of tropical zooplankton (e.g., *Moina dubia*) to fish kairomones is generally small, accounting for 2%–10% of the changes in the life‐history traits, which is several times smaller than the TGP to elevated temperature (Pham et al., 2020). The different degree of TGP reflects the nature of the stressors (mode of action, Schäfer & Piggott, 2018), the magnitude of the stress level (Donelson et al., 2012), the number of exposed generations (Pham et al., 2020), and the response variables (this study).

At the sublethal level, the magnitude of TGP to MHWs was much smaller than survival. Indeed, we found consistent and clear evidence for TGP to MHW (F1‐MHW × F2‐MHW) with 5%–10% better performance of F2 *P. incicus* compared to the immediate F2 MHW exposure (F2‐MHW alone). All were substantially lower than the performance of *P. incisus* in the control temperature. The transgenerational MHW effects caused ~50% reductions in cumulative nauplii and fecal pellets; both parameters were significantly and positively correlated, suggesting that the lowered energy intake constrained reproduction. Furthermore, the lower number of nauplii hatched per F1‐MHW × F2‐MHW exposed females further contributed to the lowered cumulative nauplii, suggesting lethal transgenerational MHW effects on embryonic development (see also Grønning et al., 2019 for *Pseudodiaptomus annandalei*). Similar effects on lowered cumulative nauplii and fecal pellets have been previously observed in *P. incisus* that was exposed to an MHW for two generations (Dinh et al., 2021). Reduced F2 *P. incisus* reproduction was likely a direct cost of maintaining survival along with some selection for tolerant genotypes from the F1 generation.

Similar to the effects on survival, the presence of FPC in F1 (F1‐FPC) or F2 (F2‐FPC) generally only played a minor role in shaping the parental MHW effect (F1‐MHW) or immediate F2 MHW effect (F2‐MHW) on the F2 generation. This was indicated by the change in the size of parental and transgenerational MHW effects, which was typically smaller than 5% of the total effects of both MHW and FPC. The dominant effect of MHW is likely widespread for most marine invertebrates, indicated by massive ecological consequences during MHWs (Hughes et al., 2019; Smale et al., 2019), including mass mortality of various groups of coastal aquatic taxa (Garrabou et al., 2009, 2019).

### 4.4 Conclusions and perspectives

There is a great concern that the hyperdiverse tropical ecosystems may be collapsing under the cumulative impact of multiple stressors (Barlow et al., 2018; Dinh, 2019; Worm et al., 2006). Among others, MHWs are becoming a severe threat, increasingly causing mass and widespread mortality across taxa (Garrabou et al., 2009, 2019). However, we still know little about how MHWs may affect key grazers and intermediate prey in coastal marine ecosystems, such as copepods, particularly in ecologically relevant contexts, e.g., with biotic interactions (Truong et al., 2020). Our results contribute to this research gap by highlighting the severe effect of MHWs on the reproduction and grazing of *P. incisus*, a common copepods species in the tropical coastal ecosystems of Southeast Asia. Strong mortality together with reduced reproductive success is an important mechanism underlying the reduced copepod abundance under MHWs (Evans et al., 2020). Consequently, MHWs may substantially reduce the secondary biomass production of zooplankton which fuels resources and energy to the vast majority of marine predators such as corals, crustaceans, and fish (Arimitsu et al., 2021; Chew et al., 2012). Most importantly, our results suggest that *P. incisus* may evolve rapidly and complete TGP to MHW, with full amelioration of its lethal effect in the second generation. The transgenerational plasticity of MHWs comes with a cost of reduced reproduction, and grazing may be a crucial and widespread mechanism for marine invertebrates coping with the transient effects of heatwaves (Cavieres et al., 2020; Dinh et al., 2020; Doan et al., 2019). Interestingly, fish predator cues played a minor role in shaping both immediate and transgenerational effects of MHWs, highlighting the dominant effects of MHWs on *P. incisus*.

MHWs occur randomly across space and time and do not provide any reliable signal for long‐term directional selection and evolution of thermal tolerance which may enable copepods to deal with the thermal stress relating to annual variations in temperatures across seasons, e.g., in temperate calanoid copepods (Sasaki & Dam, 2020). Given the increasingly severe and widespread occurrence of MHWs in coastal ecosystems, the duration and the frequency of MHWs may shape the thermal tolerance of tropical copepods in the warm season. The dominant effects of MHWs we observed in this study have important implications for ecological risk assessments in the rapidly changing environment of tropical coastal ecosystems.

## AUTHOR CONTRIBUTIONS

**Kiem N. Truong:** Conceptualization (lead); investigation (equal); methodology (equal); supervision (lead); visualization (equal); writing – original draft (lead); writing – review and editing (equal). **Ngoc‐Anh Vu:** Conceptualization (equal); data curation (lead); formal analysis (equal); investigation (lead); methodology (equal); validation (equal); visualization (equal); writing – original draft (equal); writing – review and editing (equal). **Nam X. Doan:** Conceptualization (lead); funding acquisition (lead); investigation (lead); project administration (lead); resources (lead); supervision (lead); writing – original draft (supporting); writing – review and editing (supporting). **Canh V. Bui:** Data curation (supporting); investigation (equal); methodology (supporting); supervision (supporting); writing – original draft (supporting). **Minh Hoang Le:** Conceptualization (equal); funding acquisition (equal); project administration (lead); supervision (supporting); writing – original draft (equal); writing – review and editing (equal). **Minh T.T. Vu:** Conceptualization (equal); formal analysis (equal); funding acquisition (equal); methodology (equal); writing – original draft (equal); writing – review and editing (equal). **Khuong V Dinh:** Conceptualization (lead); investigation (supporting); methodology (lead); resources (lead); supervision (lead); visualization (equal); writing – original draft (lead); writing – review and editing (lead).

## CONFLICT OF INTEREST

No conflict of interest to declare.

## Data Availability

All original datasets used in this study are available at the Figshare with the DOI accession number: https://doi.org/10.6084/m9.figshare.20294073.
